# A138 EVALUATING THE ACCEPTABILITY AND EFFICACY OF CYTOSPONGE FOR BARRETT’S ESOPHAGUS: A SINGLE CENTRE CROSS-SECTIONAL STUDY

**DOI:** 10.1093/jcag/gwae059.138

**Published:** 2025-02-10

**Authors:** A AlQattan, M Ten-Pow, L T’ien, M Choi, B Chan, C Galorport, R Enns

**Affiliations:** Gastroenterology, The University of British Columbia, Vancouver, BC, Canada; Gastroenterology, The University of British Columbia, Vancouver, BC, Canada; Gastroenterology, The University of British Columbia, Vancouver, BC, Canada; Gastroenterology, The University of British Columbia, Vancouver, BC, Canada; Gastroenterology, The University of British Columbia, Vancouver, BC, Canada; Gastroenterology, The University of British Columbia, Vancouver, BC, Canada; Gastroenterology, The University of British Columbia, Vancouver, BC, Canada

## Abstract

**Background:**

Barrett’s Esophagus (BE) is a pre-malignant condition defined by the presence of metaplastic columnar epithelial cells above the gastroesophageal junction. Once metaplasia is present, there is a 0.5% annual risk of progression to dysplasia and ultimately adenocarcinoma. Cytosponge is a new device and technique to diagnose BE. Furthermore, this test has a strong safety profile. Research showed increased patient tolerance for the Cytosponge compared to endoscopy; however, this has not been demonstrated in a Canadian setting. Ideally, patient acceptability of Cytosponge device should be evaluated before its integration in a Canadian context.

**Aims:**

To assess patient acceptability, tolerability and integration of Cytosponge in the diagnosis of Barrett’s Esophagus in a Canadian healthcare setting. We also assessed the ease of use and familiarity with Cytosponge.

**Methods:**

A single-centre, prospective cross-sectional study was conducted to evaluate the acceptability and comfort of patients undergoing Cytosponge procedure. Outpatients referred for EGD for Barrett’s Esophagus at St. Paul’s Hospital between 03/21-08/24 were included. 74 patients with BE have been enrolled in this project. Acceptability was evaluated through Visual Analogue Scale (VAS), Spielberger State Trait Anxiety Inventory (STAI), and Impact of Events Scale (IOES) on the day of procedure, day 7, and day 90 post-procedure. Data from health care providers administering the Cytosponge were collected using the System Usability Scale (SUS). One-way ANOVA and Tukey’s Honestly Significant Difference tests were completed to assess score differences between follow-ups.

**Results:**

A total of 74 patients met the inclusion criteria and consented. 89.2% were successful in swallowing Cytosponge, 10.8% were unsuccessful. ANOVA test were statistically significant in VAS scores between day 0 and day 7, day 0 and day 90 with a p-value 0.006 and 0.00005 respectively. Average STAI scores pre- and post-Cytosponge were 41.7 (SD 3.1) and 40.7 (SD 4.4), ANOVA analysis of STAI scores between groups showed no statistical significance. The average IOES score at day 0 was 1.7 (SD 3.4), day 7 1.1, and day 90 0.7. There was no statistically significant difference in IOES scores at follow-up.

**Conclusions:**

Our results demonstrate that Cytosponge is well tolerated in a Canadian healthcare setting. Average STAI and IOES scores were comparable at follow up. Additionally they were not statistically significant suggesting that patients found Cytosponge an acceptable. A score of 68 and above is above average on SUS which measures usability of Cytosponge. The average SUS score in this sample was 55.2 this suggests that there is a learning curve for healthcare providers to become familiar with Cytosponge. There were no complications with Cytosponge in this sample.

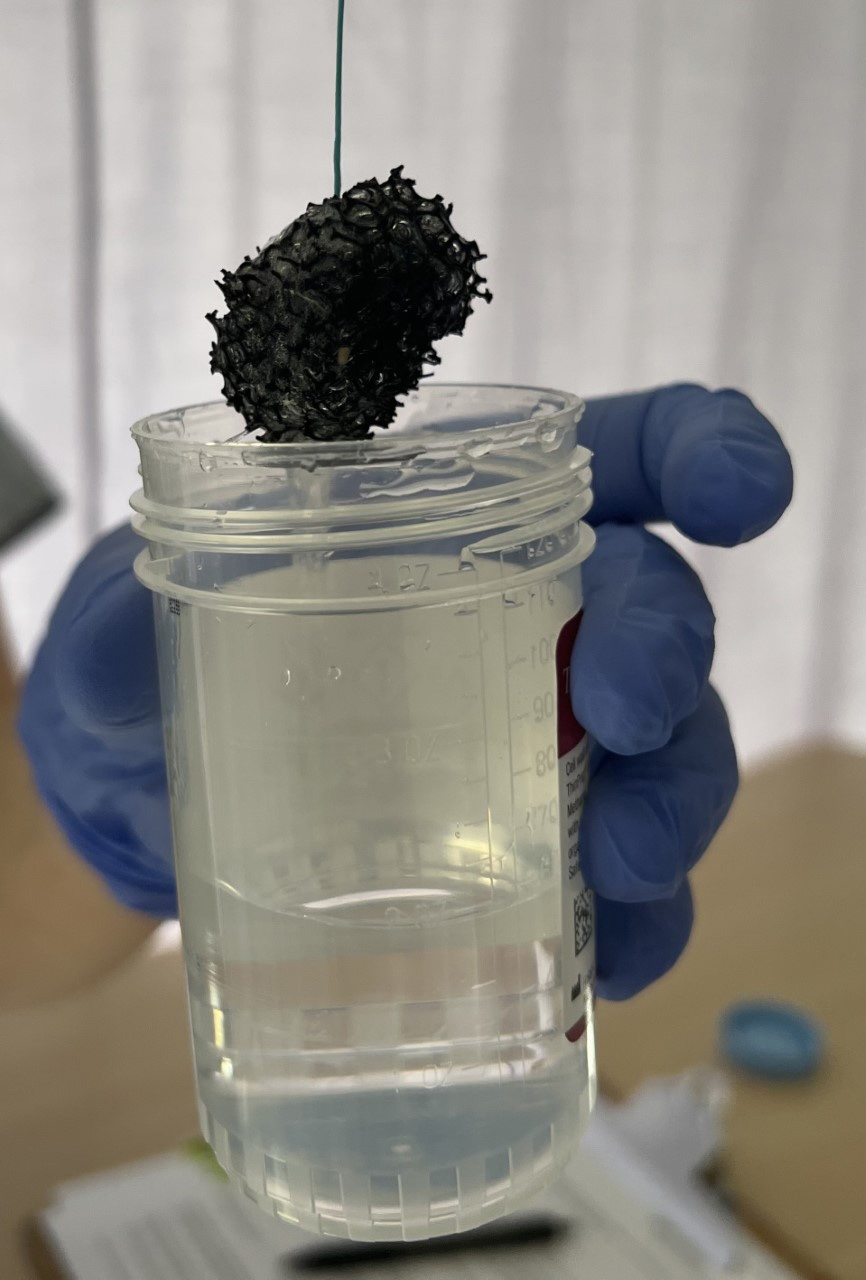

Expanded and retrieved Cytosponge device

**Funding Agencies:**

Gastroenterology Institute of Research Institute

